# The effect of Rbfox2 modulation on retinal transcriptome and visual function

**DOI:** 10.1038/s41598-020-76879-5

**Published:** 2020-11-12

**Authors:** Lei Gu, Riki Kawaguchi, Joseph Caprioli, Natik Piri

**Affiliations:** 1 Stein Eye Institute, University of California, Los Angeles, 100 Stein Plaza, Los Angeles, CA 90095 USA; 2 Department of Psychiatry and Neurology, University of California, Los Angeles, Los Angeles, CA 90095 USA; 3 Brain Research Institute, University of California, Los Angeles, Los Angeles, CA 90095 USA

**Keywords:** Cell biology, Molecular biology, Neuroscience

## Abstract

Rbfox proteins regulate alternative splicing, mRNA stability and translation. These proteins are involved in neurogenesis and have been associated with various neurological conditions. Here, we analyzed Rbfox2 expression in adult and developing mouse retinas and the effect of its downregulation on visual function and retinal transcriptome. In adult rodents, Rbfox2 is expressed in all retinal ganglion cell (RGC) subtypes, horizontal cells, as well as GABAergic amacrine cells (ACs). Among GABAergic AC subtypes, Rbfox2 was colocalized with cholinergic starburst ACs, NPY (neuropeptide Y)- and EBF1 (early B-cell factor 1)-positive ACs. In differentiating retinal cells, Rbfox2 expression was observed as early as E12 and, unlike Rbfox1, which changes its subcellular localization from cytoplasmic to predominantly nuclear at around P0, Rbfox2 remains nuclear throughout retinal development. *Rbfox2* knockout in adult animals had no detectable effect on retinal gross morphology. However, the visual cliff test revealed a significant abnormality in the depth perception of *Rbfox2*-deficient animals. Gene set enrichment analysis identified genes regulating the RNA metabolic process as a top enriched class of genes in *Rbfox2*-deficient retinas. Pathway analysis of the top 100 differentially expressed genes has identified Rbfox2-regulated genes associated with circadian rhythm and entrainment, glutamatergic/cholinergic/dopaminergic synaptic function, calcium and PI3K-AKT signaling.

## Introduction

The family of RNA binding protein, fox-1 (Rbfox) homolog includes three evolutionarily conserved members, Rbfox1, Rbfox2 and Rbfox3, that regulate cell- or tissue-specific alternative splicing at different developmental stages. Alternative splicing is an important mechanism to generate greater proteomic diversity from a fixed genome; disruptions of normal programs of splicing regulatory networks have been associated with various human diseases, including neurological disease^1^. Rbfox proteins have a single RNA recognition motif (RRM) type RNA binding domain that facilitates its association with the *(U)GCAUG* element located in alternatively spliced exons or in flanking introns. Typically, binding of Rbfox downstream of the alternative exon promotes its splicing, whereas binding to an upstream element, or an element within the exon, represses exon inclusion. High-throughput sequencing of RNA isolated by crosslinking immunoprecipitation (HITS-CLIP) and integrative modeling defined 1059 direct Rbfox target alternative splicing events that regulate global dynamic splicing changes during mouse brain development^2^. Rbfox is a family of multifunctional proteins; in addition to regulating alternative splicing, they control mRNA stability and translation efficiency^3^. For instance, Rbfox2 mediates transcriptional repression via interaction with chromatin‐associated nascent RNA, often near gene promoters and its functional interplay with polycomb repressive complex 2, which methylates histone H3 and is required for epigenetic gene silencing^4^. The functional diversity of Rbfox proteins in regulating RNA metabolism is supported by the expression of number of isoforms for each family member^3,5^. For instance, alternatively spliced mouse Rbfox1 without exon 19 generates a nuclear isoform with a nuclear localization signal (NLS). Rbfox1 isoform with exon 19 has no NLS and consequently localizes to the cytoplasm. Nuclear Rbfox1 regulates splicing events, whereas the cytoplasmic isoform is associated with mRNA stability and translation efficiency^6^.

Our interest in expression and function of *Rbfox* genes in the retina originates from the study that was designed to obtain and characterize the retinal ganglion cell (RGC) transcriptome^7^. RGC transcriptome analyses can provide important information about normal RGC function, the differences between RGC subtypes, and the pathophysiology of optic neuropathies associated with RGC degeneration. Ganglion cells are the projection neurons of the retina; they receive visual information from photoreceptors via bipolar and amacrine cells (AC) and convey this information to postsynaptic targets in the brain. Transcriptome and functional classification of mouse RGCs identified 40 and at least 32 groups of RGCs, respectively^8,9^. The dendritic arbors of each RGC subtype tile the entire retinal surface so specific features of the image can be detected and communicated to the brain^10^. Dysfunction and degeneration of RGCs and their axons in the optic nerve lead to loss of vision in various optic neuropathies. The most common form of optic neuropathies, glaucoma, affects more than 70 million people worldwide and is the leading cause of irreversible blindness^11^.

Investigating Rbfox proteins in the retina is of particular interest to us since they are known to regulate extensive genetic networks in developing and mature neurons. Mutations and deficiencies in Rbfox gene expression have been associated with several neurological conditions including autism spectrum disorder (ASD), mental retardation, epilepsy, ADHD, bipolar disorder, schizoaffective disorder and schizophrenia^12–15^. Recently, we reported our findings on the expression pattern of Rbfox1 in the retina, evaluated changes in the retinal transcriptome and visual function in response to Rbfox1 downregulation^16^. The current work is a continuation of our study on the role of Rbfox proteins in the retina and presents data on the comprehensive analysis of Rbfox2 expression in differentiating and adult mouse retinas, as well as the effect of *Rbfox2* downregulation on the retinal transcriptome and on visual function such as pupillary light reflex and depth perception.

## Results

### Expression of Rbfox2 in adult and developing retinas

#### Rbfox2 is expressed in RGCs, ACs and horizontal cells (HCs) of adult mouse retinas

**Figure 1:**
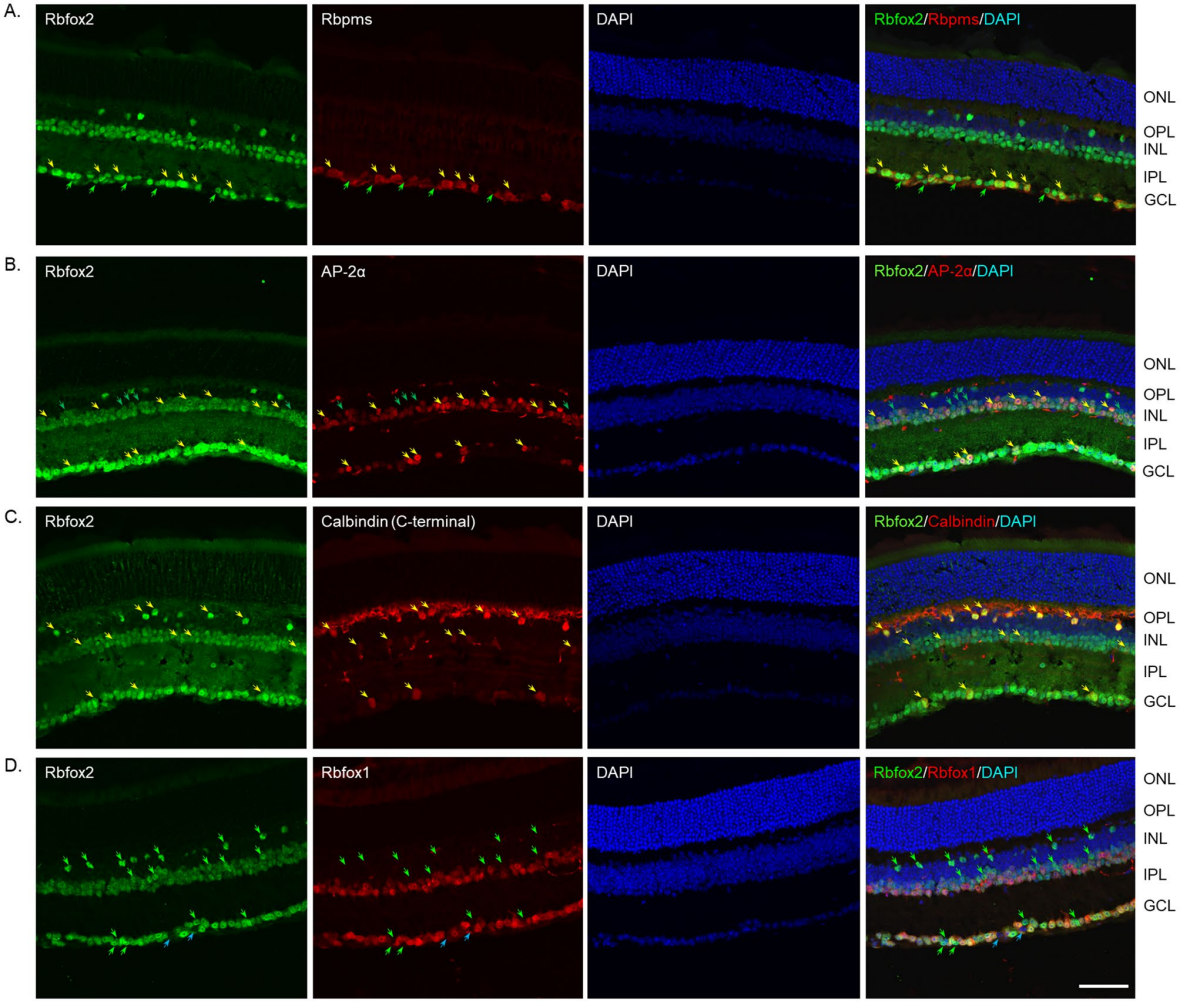
Rbfox2 expression in adult mouse retina. Rbfox2 immunoreactivity was present in the ganglion cell layer (GCL) and inner nuclear layer (INL) of the retina. In the INL, which contains cell bodies of horizontal cells (HCs), bipolar cells and amacrine cells (ACs), as well as Muller glia cells, Rbfox2^+^ cells were primarily localized in 2–3 rows of cells proximal to the inner plexiform layer (IPL) and in sparsely distributed cells adjacent to outer plexiform layer (OPL). (**A**) In the GCL, Rbfox2 was colocalized with Rbpms^+^ RGCs (yellow arrows). Green arrows point at Rbfox2^+^/Rbpms^−^. (**B**) Virtually all AP-2α^+^ cells (AP-2α is an AC marker) were Rbfox2^+^ (yellow arrows). Very few Rbfox2^+^ cells were AP-2α^−^ (green arrows). (**C**) Rbfox2 was also expressed in calbindin^+^ HCs (yellow arrows). (**D**) A significant overlap in Rbfox1 and Rbfox2 expression within INL and GCL was observed. However, Rbfox2^+^ cells were more widely distributed among ACs in the INL and GCL than Rbfox1^+^ cells. Furthermore, Rbfox2 but not Rbfox1 was expressed in HCs. Green arrows point at Rbfox2^+^/Rbfox1^−^ cells in the GCL and INL. Very few cells in the GCL appeared to be Rbfox2^−^ (blue arrows). ONL, outer nuclear layer, DAPI; 4′,6-diamidino-2-phenylindole. Scale: 50 µm.

**Figure 2:**
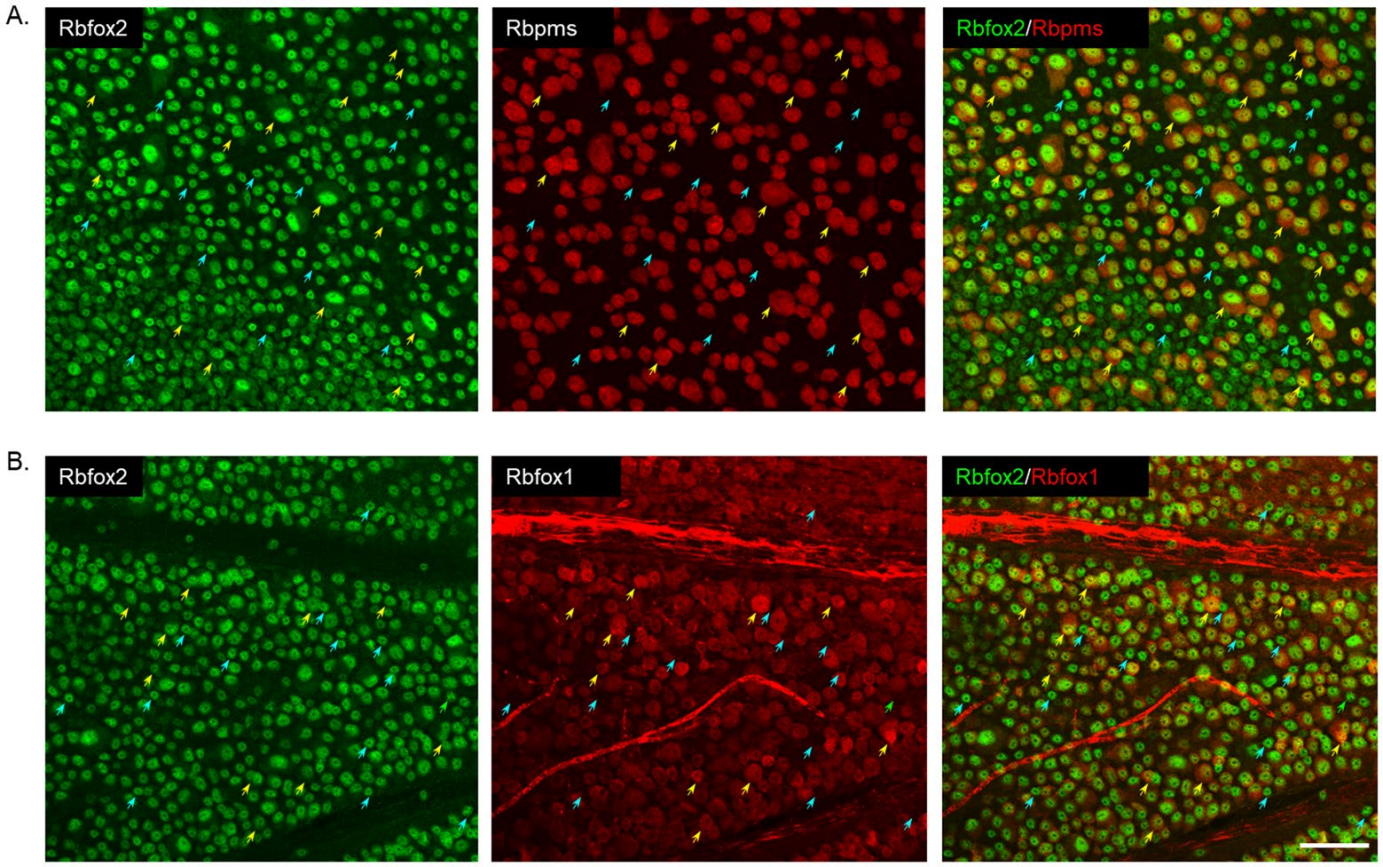
Colocalization of Rbfox2 with Rbpms and Rbfox1 in whole mount retina. (**A**) Rbfox2 was expressed in all Rbpms^+^ RGCs (yellow arrows). Rbfox2^+^/Rbpms^−^ cells (dACs) are indicated by blue arrows. (**B**) A significant overlap in Rbfox1 and Rbfox2 expression was observed (yellow arrows point at some Rbfox1^+^/Rbfox2^+^ cells). However, approximately 6–7% of cells express Rbfox2 but not Rbfox1 (blue arrows). Scale: 50 µm.

The spatial expression pattern of Rbfox2 in adult mouse retinas was analyzed by immunohistochemistry with anti-Rbfox2 antibodies. Rbfox2 immunostaining was restricted to cells located in the ganglion cell layer (GCL) and inner nuclear layer (INL) of the retina (Fig. 1). Double immunostaining with Rbfox2 and RGC marker Rbpms showed that virtually all Rbpms-labeled RGCs are also Rbfox2^+^ (Fig. 1A; pointed with yellow arrows). In addition to Rbfox2^+^ RGCs, there were Rbfox2-stained cells in the GCL that were not labeled with anti-Rbpms antibodies suggesting that Rbfox2 is also expressed in displaced ACs (dACs; Fig. 1A; pointed with green arrows). The GCL in rodent retinas contains two types of neurons, RGCs and displaced ACs (dACs), in a ratio of approximately 1:1: ~ 45% RGCs and ~ 55% dACs are present in mouse retinas; in rat retinas, these cells are in ~ 50:50 ratio; and in hamster retinas, there are 56% RGCs and more than 40% dACs^17–20^. In the INL, which contains cell bodies of horizontal cells (HCs), bipolar cells and ACs, as well as Muller glial cells, Rbfox2^+^ cells were mostly localized to two rows of cells proximal to the inner plexiform layer (IPL). ACs of the mouse retina form two to four rows of cells at the inner margin of the INL^17,21^, suggesting that Rbfox2^+^ cells in the INL are ACs. Indeed, these cells were colocalized with AP-2α, an AC marker^22^; virtually all AP-2α^+^ cells were also stained for Rbfox2 (Fig. 1B; pointed with yellow arrows). However, there were very few Rbfox2^+^ cells that were AP-2α^−^ (Fig. 1B; pointed with green arrows). Furthermore, Rbfox2 was also expressed in sparsely distributed cells in the INL that were adjacent to the outer plexiform layer (OPL). These cells were colocalized with calbindin^+^ HCs (Fig. 1C; pointed with yellow arrows). Calbindin is commonly used as a marker for both ACs and HCs in the mouse retina. The anti-calbindin antibodies generated against calbindin C-terminal peptide that we used in this immunostaining appears to label HCs much stronger than ACs^23,24^. Double staining with antibodies against Rbfox2 and Rbfox1 was performed to evaluate the extent of potential overlap in expression of Rbfox2 and Rbfox1 in the retina (Fig. 1D; pointed with green arrows). Many cells in the INL and GCL express both isoforms, however significant differences are evident: Rbfox2 but not Rbfox1 is expressed in HCs and there is a wider distribution of Rbfox2^+^ cells particularly in the INL. There are also cells in the GCL that express only Rbfox2 and, since all RGCs express Rbfox1^16^, these Rbfox2^+^ cells must be subtypes of dACs (Fig. 1D; pointed with green arrows). It should be noted that typically all GCL cells are Rbfox2^+^, however, in some retinal sections we occasionally see cells in the GCL that are Rbfox2^−^ (Fig. 1D; pointed with blue arrows). Since retinal sections represent a small portion of the tissue and may not accurately represent the entire retina, whole mounted retinas were used for quantitative analysis of Rbfox2^+^ cells. Rbfox2^+^/Rbpms^+^ and Rbfox1^+^/Rbfox2^+^ cells were counted in the superior, inferior, nasal and temporal quadrants of the retinas 1 mm from the center of the optic nerve (Fig. 2). Almost half of Rbfox2^+^ cells (868.67 ± 25.08 cells/mm^2^) were colocalized with Rbpms (4200 ± 24.99 cells/mm^2^). Virtually 100% of RGCs (Rbpms^+^ cells) were Rbfox2^+^ (Fig. 2A). Colocalization of Rbfox1^+^ and Rbfox2^+^ cells showed extensive overlap in their expression in the GCL but approximately 6–7% of cells express only Rbfox2 (Fig. 2B).

#### Rbfox2 is expressed in GABAergic ACs

**Figure 3:**
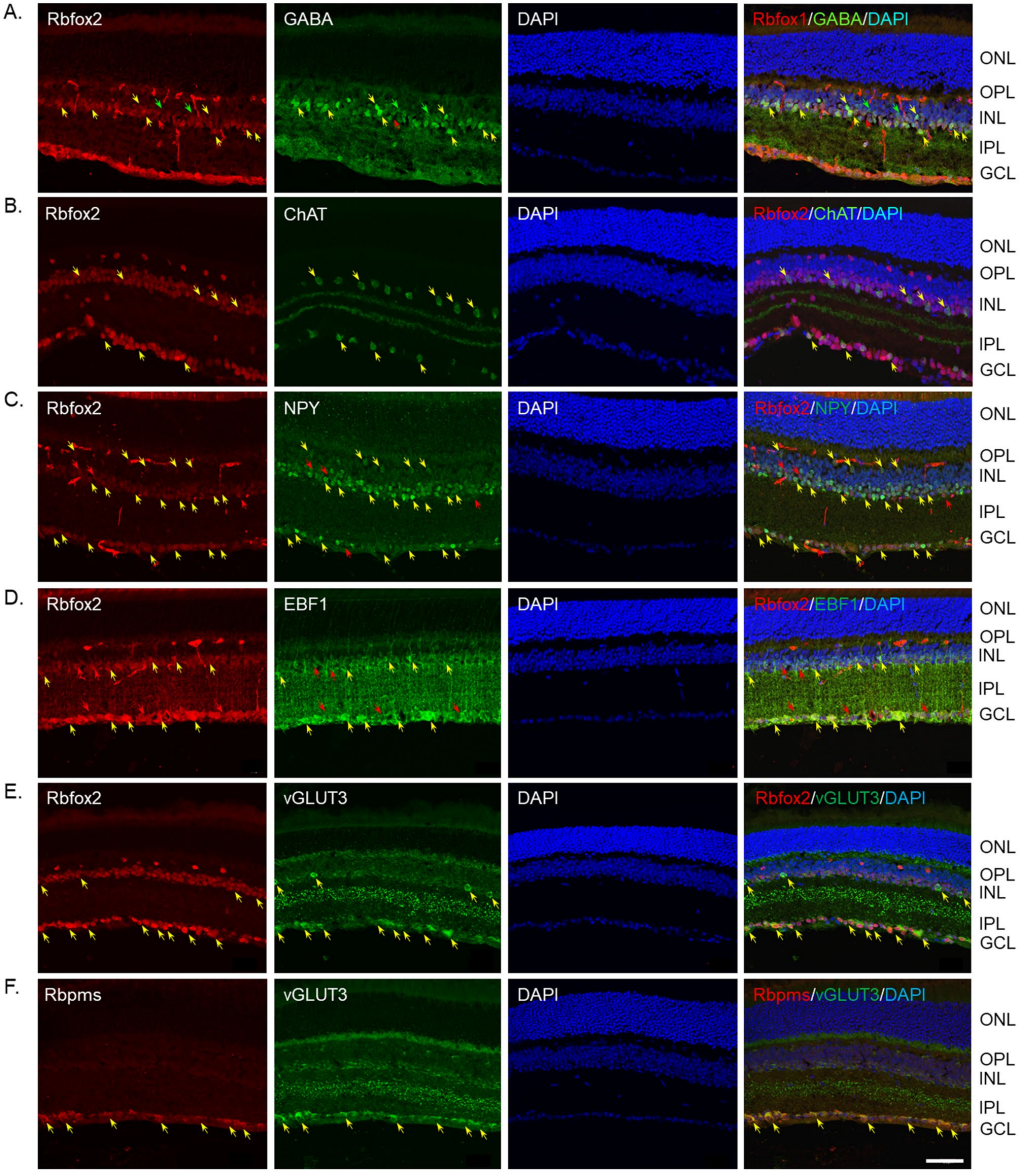
Localization of Rbfox2 expression in AC subtypes. Several markers of AC subtypes, including GABA, choline acetyltransferase (ChAT), NPY, early B-cell factor 1 (EBF1), glycine transporter (GlyT1) and vesicular glutamate transporter 3 (vGlut3) were used to determine the identity of Rbfox2^+^ ACs. (**A**) The vast majority GABAergic ACs in the INL were Rbfox2^+^. Some GABA^+^/Rbfox2^+^ ACs pointed by yellow arrows. Green arrows point at Rbfox2^−^ GABAergic ACs. In the GCL, all GABAergic ACs were also Rbfox2^+^. (**B**) Rbfox2 was expressed in all cholinergic (ChAT^+^) starburst ACs (SACs) both in the INL and GCL. Extensive overlap of Rbfox2 with NPY (**C**) and EBF1 (**D**) expression was also observed (yellow arrows), although there were very few Rbfox2^+^ cells that had very faint or no staining for NPY or EBF1 (pointed by red arrows). (**E**) Very few vGlut3^+^ ACs were detected in the INL and they were also Rbfox2^+^. vGlut3^+^ cells were present in abundance in the GCL. These cells were colocalized with Rbpms^+^ RGCs (**F**). Scale: 50 µm.

ACs are inhibitory interneurons, which represented in the mammalian retina by morphologically and functionally diverse cell types. Based on the type of neurotransmitter that ACs synthesize and use for their function, they can be classified into two groups: wide-field ACs that express gamma-aminobutyric acid (GABA) and narrow-field ACs that express glycine^25,26^. GABA and glycine considered as the primary neurotransmitters of ACs. GABAergic and glycinergic ACs can be further subdivided into subgroups that synthesize other neuromodulators including acetylcholine, dopamine, somatostatin, substance P, neuropeptide Y (NPY), serotonin vasoactive intestinal polypeptide, and endocannabinoids. We used several markers of ACs, including GABA, choline acetyltransferase (ChAT), NPY, early B-cell factor 1 (EBF1), glycine transporter (GlyT1) and vesicular glutamate transporter 3 (vGlut3) to define the subtype identity of Rbfox2 + ACs. The vast majority of GABAergic ACs in the INL were Rbfox2 + (Fig. 3A; yellow arrows point at GABAergic ACs that express Rbfox2; green arrows point at Rbfox2-negative ACs). It appears that almost all Rbfox2^+^ cells in the INL were GABAergic, but some GABAergic ACs were Rbfox2^−^ (Fig. 3A; GABA^+^/Rbfox2^−^ cells are pointed by green arrows). In the GCL, all GABAergic ACs were also Rbfox2^+^. A subtype of GABAergic ACs, cholinergic (ChAT^+^) starburst ACs (SACs), were all Rbfox2^+^ in both INL and GCL (Fig. 3B). Extensive overlap of Rbfox2 with NPY and EBF1 expression was also observed, although there were very few Rbfox2^+^ cells that had very faint or no staining for NPY or EBF1 (Fig. 3C,D). Very few vGlut3^+^ ACs were detected in the INL: they were Rbfox2^+^ (Fig. 3E). vGlut3^+^/Rbfox2^+^ cells were present in abundance in the GCL. However, these cells were not ACs: they were Rbpms^+^ RGCs (Fig. 3F).

#### Rbfox2 expression during retina development

**Figure 4:**
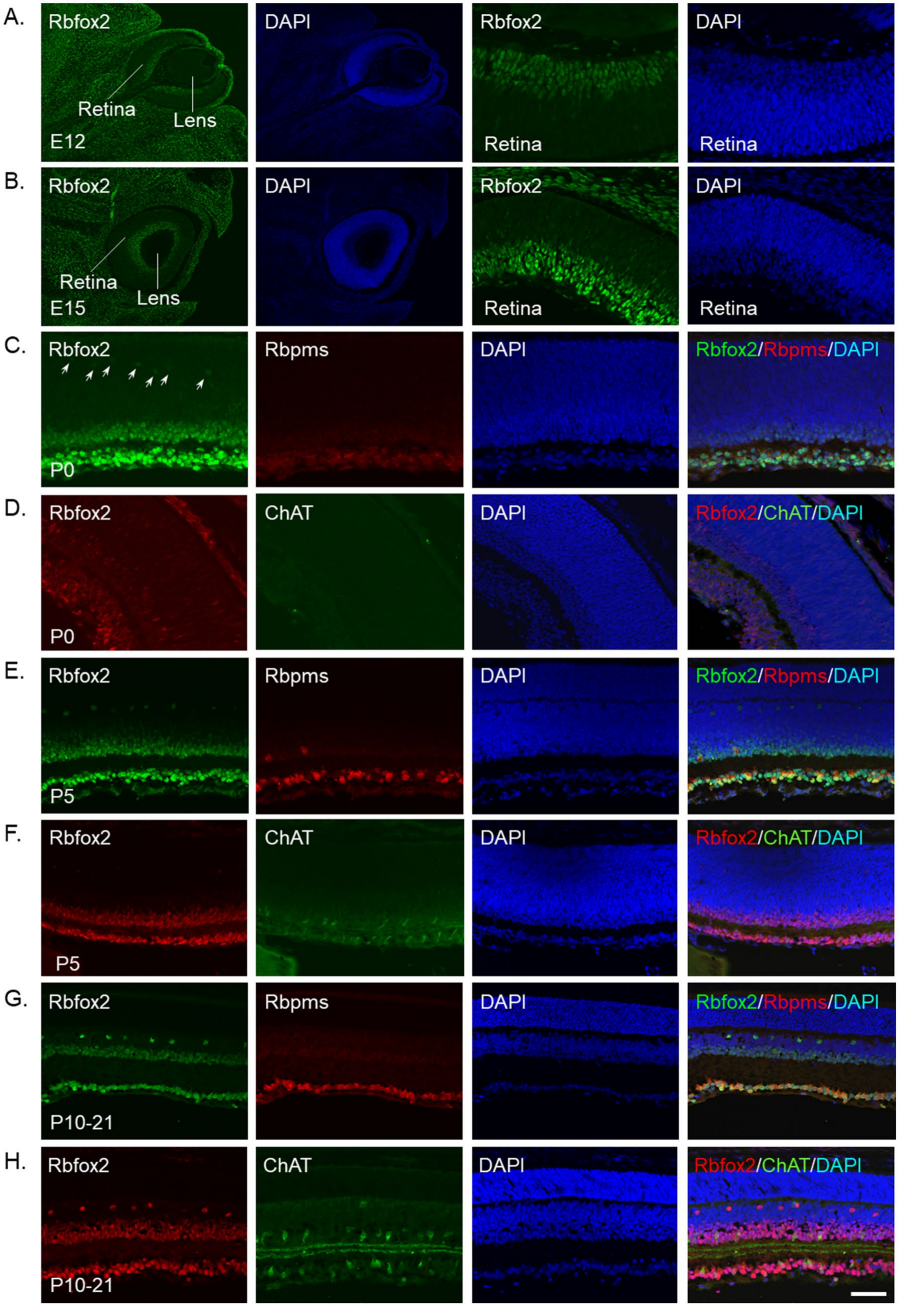
Expression of Rbfox2 during retina differentiation. Mouse retinas at E12, E15, P0, P5, P10, P15 and P21 were used. (**A**,**B**) Rbfox2 immunostaining in differentiating retinas was detected as early as E12. The pattern of Rbfox2 expression at E15 was similar to that at E12, although an increase in its staining intensity level was observed. Two right images show Rbfox2 expression in the entire developing eye (the locations of the retina and the lens are indicated). Two left images show Rbfox2 expression in the retina at higher magnification. (**C**) At P0, the IPL clearly separates, GCL from the rest of the retina. Rbfox2 staining was observed in the GCL and in several rows of cells above the IPL. At this stage of retinal cell differentiation, weakly stained Rbfox2^+^ cells, presumably HCs based on their location and distribution in the retina, were detected (white arrows). Rbfox2^+^ cells in the GCL were colocalized with Rbpms^+^ cells. (**D**) No staining for ChAT was detected at P0. (**E,F**) At P5, a narrow OPL that separates ONL from INL was apparent. First ChAT^+^ cells were detected at P5. (**G,H**) At P10, P15 and P21, Rbfox2 expression patterns were similar to that of fully differentiated retina. Both Rbpms^+^ and ChAT^+^ cells were also Rbfox2^+^. Scale: 50 µm.

Proteins of Rbfox family have been associated with neurogenesis and synaptogenesis^27,28^ and, therefore, we evaluated expression of these proteins during retinal development. In mouse retinas, the first cell types to differentiate from multipotent progenitor cells are RGCs and HCs, followed in overlapping phases by cone photoreceptors, ACs, rod photoreceptors, bipolar cells and, finally, Müller glia cells^29^. Expression of Rbfox2 was present starting on E12, the earliest stage that we used in this study (Fig. 4A). No significant difference in Rbfox2 expression pattern between E12 and E15 was observed, except a noticeable increase in its staining intensity level at the later stage (Fig. 4B). At P0, the IPL, which separates GCL from the rest of the retina can be seen (Fig. 4C). At this stage, Rbfox2 staining was present in the GCL as well as in several rows of cells above the IPL. Furthermore, faintly stained Rbfox2^+^ cells that were most likely HCs were present. At P5, a narrow OPL that separates ONL from INL was apparent (Fig. 4E). Rbfox2 staining patterns at P5, P10, P15, P21 and in the mature retina were very similar: its expression was restricted to dACs and RGCs within the GCL and to HCs, and to first three rows of ACs adjacent to the IPL within the INL (Fig. 4E). Colocalization of Rbfox2 with Rbpms-labeled RGCs and ChAT^+^ SACs are shown in Fig. 4E–H. The earliest stage that Rbpms staining can be reliably observed is at P0 (Fig. 4C). Rbpms staining in P5 and older retinas becomes more prominent and defined. With respect to SACs, which were reported to be the first AC subtype to differentiate from retinal progenitor cells (50% of ChAT^+^ ACs are born between E8 and E14)^21^, we were able to identify the first ChAT^+^ cells with confidence at P5 (Fig. 4D,F). Both Rbpms^+^ and ChAT^+^ cells were also Rbfox2^+^ at all stages of retinal differentiation.

### Histological, behavioral and functional assessment of Rbfox2^−/−^ animals

#### *Rbfox2* downregulation has no recognizable effect on retinal architecture

**Figure 5:**
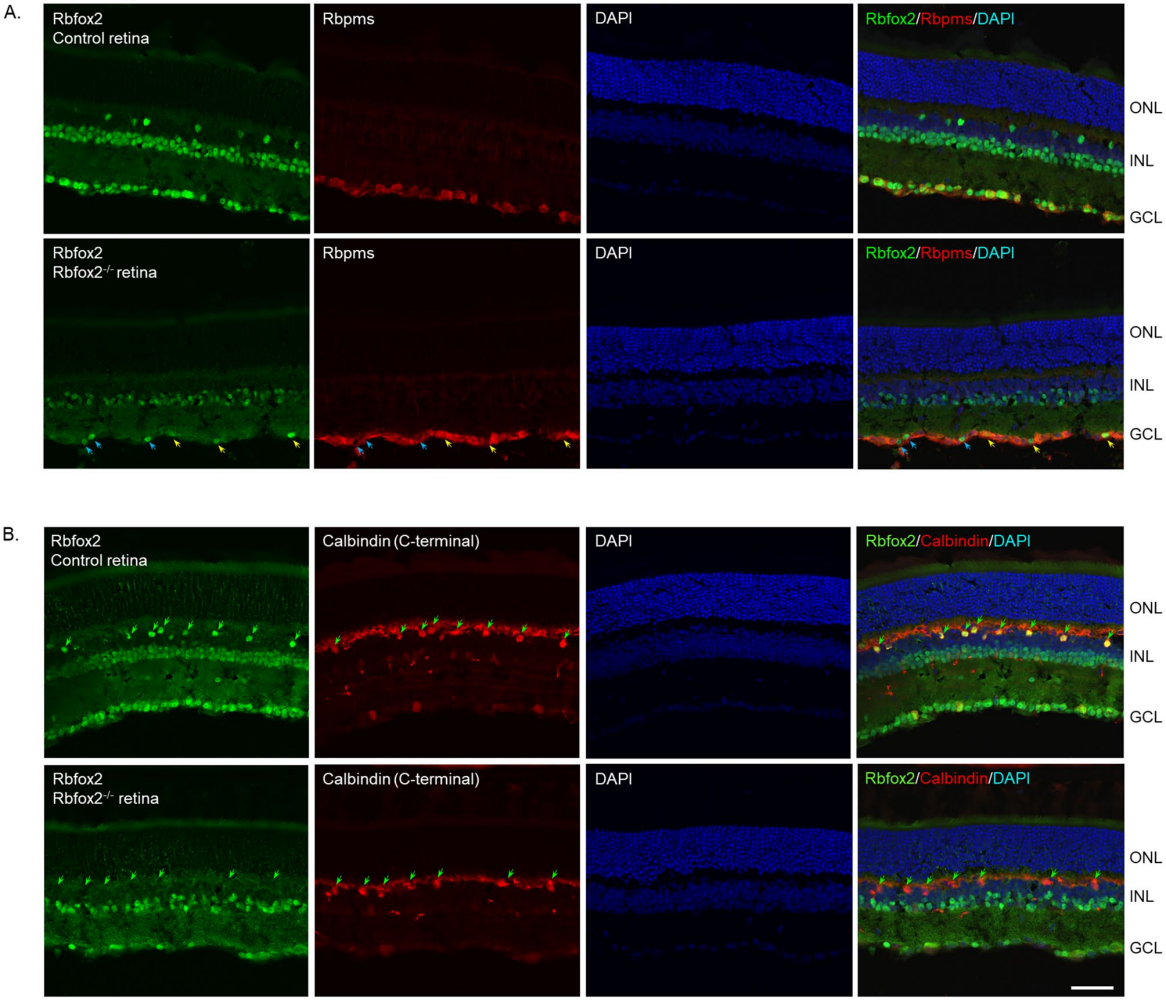
Histological evaluation of retinal architecture in *Rbfox2*^*−/−*^ animals. Control and *Rbfox2*^*−/−*^ retinal sections were immunostained with antibodies against Rbfox2 and Rbpms (**A**) and Rbfox2 and calbindin (**B**). Very few Rbfox2^+^ cells were present in the GCL of Rbfox2^−/−^ animals. Staining for Rbpms and calbindin appears to be not affected by *Rbfox2* downregulation. Colocalization of remaining Rbfox2^+^ cells in the GCL with Rbpms showed that downregulation of this gene in *Rbfox2*^*−/−*^ mice is taking place in both RGCs and dACs: three out of six Rbfox2^+^ cells were colocalized with Rbpms (yellow arrows) indicating that these cells are RGCs and the remaining 3 are dACs (blue arrows). Furthermore, in the INL of *Rbfox2*^*−/−*^ mice, Rbfox2 expression was significantly reduced in ACs and was completely abolished in HCs (green arrows). Retinal morphology in *Rbfox2*^*−/−*^ animals appeared to be normal. Scale: 50 µm.

*Rbfox2*^*−/−*^ animals were generated by crossing *Rbfox2*^*fl/fl*^ homozygous transgenic mice with *Tg(UBC-cre/ERT2)1Ejb* mice. The *Tg(UBC-cre/ERT2)1Ejb* mouse was used in these experiments since it shows robust Cre-mediated reporter expression in RGCs, in dACs, in ACs adjacent to the IPL and in some sparsely distributed cells in the INL adjacent to the OPL, most probably HCs (http://www.informatics.jax.org/recombinase/specificity?id=MGI:3707333&system=sensory+organs). Tamoxifen-induced Rbfox2 downregulation and its effect on retinal morphology and architecture was evaluated in adult animals four and nine months after the last administration of the drug. A dramatic reduction in Rbfox2 expression in the GCL and INL was observed (Fig. 5A,B). Very few Rbfox2^+^ cells in the GCL were detected, indicating that the downregulation of Rbfox2 effectively takes place in both RGCs and dACs. On the representative image shown in Fig. 5A, only six Rbfox2^+^ cells were present in the GCL, three of which were colocalized with Rbpms. Robust, but relatively modest compared to GCL, downregulation of Rbfox2 was observed in ACs located in the INL. Furthermore, expression of Rbfox2 was completely abolished in HCs (Fig. 5B). Despite this significant downregulation of Rbfox2, no aberrations in overall retinal gross morphology or cellular architecture were detected.

#### Pupillary light reflex (PLR)

**Figure 6:**
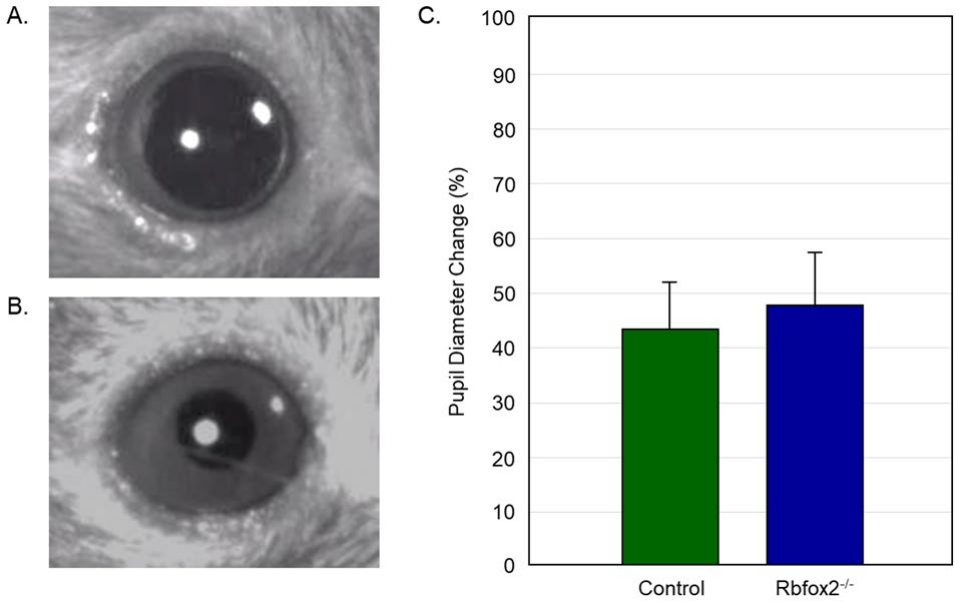
Downregulation of Rbfox2 has no detectable effect on pupillary light reflex (PLR). Representative images of dilated pupil in dark-adapted (**A**) animals and light-induced pupil constriction (**B**). (**C**) PLR appeared to be normal in *Rbfox2*^*−/−*^ mice. No statistically significant difference in maximal constriction amplitude, the time from the light onset to maximal constriction and the time from the end of light exposure to maximal pupil dilation between Rbfox2^−/−^ and control groups of animals was detected.

PLR test which measures the change in pupil diameter in response to change in light intensity was performed to evaluate the integrity of the non-image forming visual pathway mediated by M1 subtype of intrinsically photosensitive RGCs^30^. Light-dependent pupillary constriction appeared to be normal in both the control and *Rbfox2*^*−/−*^ mice (Fig. 6A–C). No statistically significant difference in maximal constriction amplitude (Fig. 6C), the time from the light onset to maximal constriction (21.75 ± 7.38 s in control vs 23.31 ± 7.48 s in *Rbfox2*^*−/−*^; p = 0.55) and the time from the end of light exposure to maximal pupil dilation (29.5 ± 7.78 s in control vs 22.31 ± 7.22 s in Rbfox2^−/−^; P = 0.46) between these groups of animals was detected.

#### Depth perception

**Figure 7:**
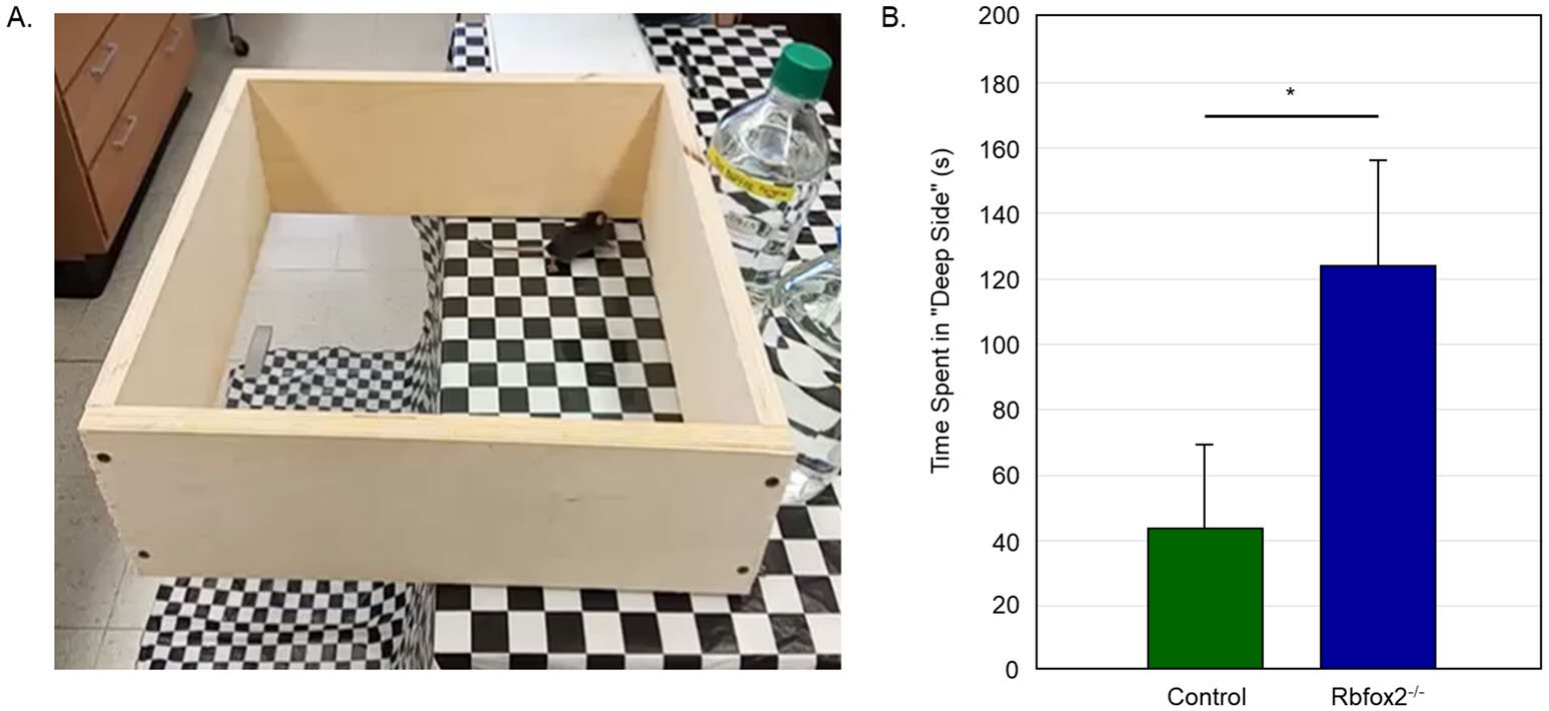
*Rbfox2*^*−/−*^ mice have deficiency in depth perception. Visual cliff test was used to evaluate depth perception in control (n = 8) and *Rbfox2*^*−/−*^ (n = 10) mice. (**A**) Visual cliff test setup. Time the animal spent on the shallow and “illusionary” deep sides during 5 min of testing was recorded. Each mouse was tested five times. (**B**) *Rbfox2*^*−/−*^ animals showed less preference for the shallow side and spent more time on the deep side of the chamber compared to controls. The overall mean (± SD) time spent on the deep side was 123  ± 34 s for animals in the *Rbfox2*^*−/−*^ group and 43 ± 24.55 s for animals in the control group, respectively. The mean difference in time spent on the deep side between two groups was statistically significant (p = 1.39372E−18).

A visual cliff test that relies on the innate tendency of animals to avoid the deep side of the field^31^ was used to evaluate visual depth perception in *Rbfox2*-deficient animals (Fig. 7A). Control mice, as expected, had a clear preference for the shallow side of the box and tried to avoid the deep side of the field. Animals with *Rbfox2* downregulation were less selective in preferring the shallow side and spent more time on the deep side than on the shallow side. The overall mean (± SD) time spent on the deep side was 123 ± 34 s for animals in the *Rbfox2*^*−/−*^ group and 43 ± 24.55 s for animals in the control group (Fig. 7B). There was a statistically significant mean difference in time spent on the deep side between two groups (p = 1.39372E−18).

#### Retinal transcriptome analysis

**Figure 8:**
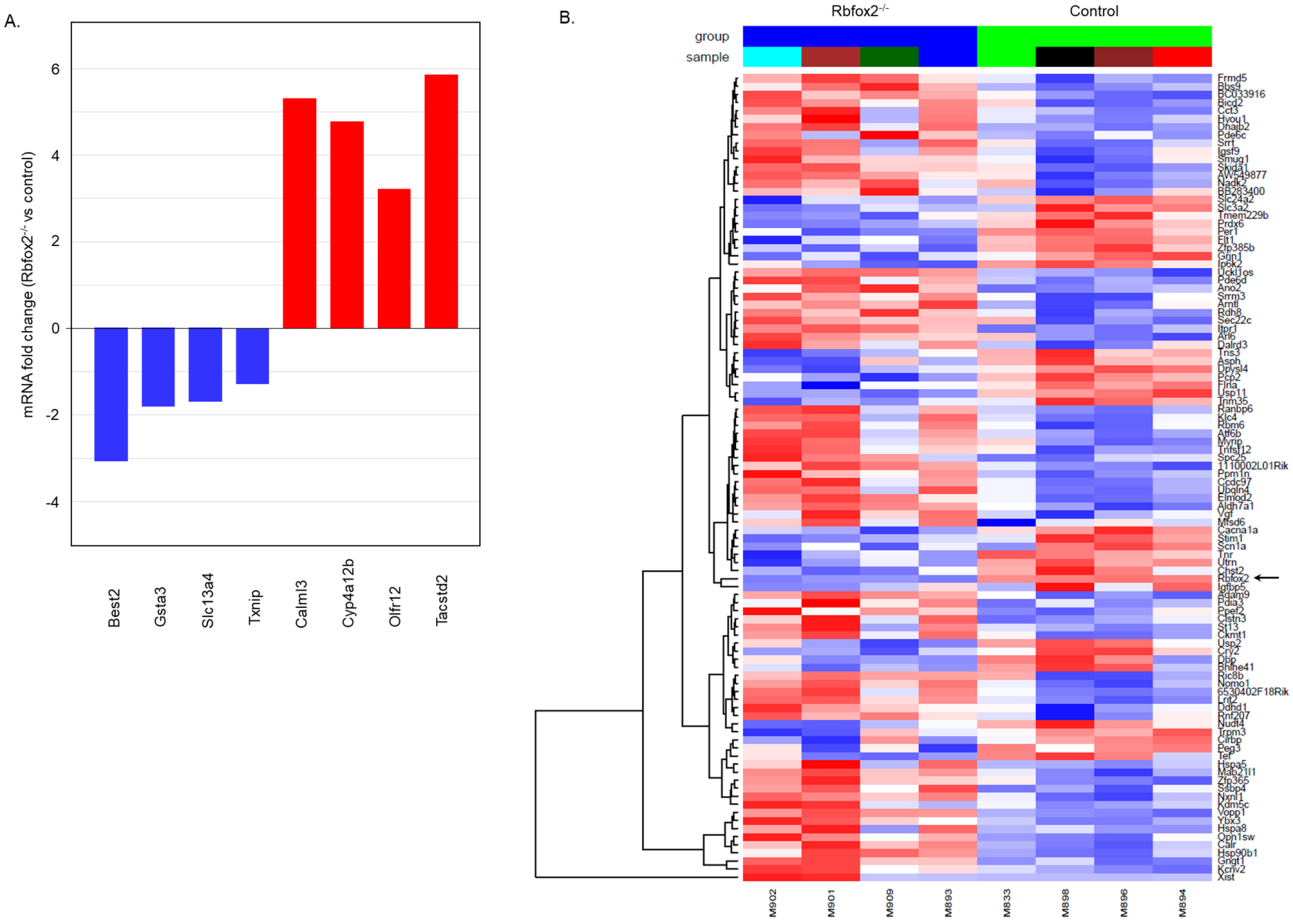
RNA-seq analysis of *Rbfox2*^*−/−*^ retinal transcriptome. (**A**) Real-time PCR quantification of several differentially regulated genes identified by RNA-seq. Downregulation of bestrophin 2 (*Best2*), glutathione S-transferase alpha3 (*Gsta3*), solute carrier family 13 member 4 (*Slc13a4*), thioredoxin interacting protein (*Txnip*) and upregulation of calmodulin like 3 (*Calml3*), cytochrome P450 monooxygenase (*Cyp4a12b*), olfactory receptor 12 (*Olfr12*) and tumor-associated calcium signal transducer 2 (*Tacstd2*) in the retinas of *Rbfox2*^*−/−*^ animals observed by quantitative real-time PCR corroborate the results of RNA-seq. (**B**) A heatmap represents the top 100 differentially expressed genes (DEG) in *Rbfox2*^*−/−*^ retinas compared to controls [EdgeR (Bioconductor 3.11; https://bioconductor.org); p < 0.01]. Four *Rbfox2*^*−/−*^ and four control animals were used for RNA-seq. Red and blue colors in the heatmap indicate over- and under-represented genes, respectively. The arrow points at *Rbfox2*, which is, as expected, was downregulated in retinas of *Rbfox2*^*−/−*^ animals.

To identify Rbfox2 regulated genes in HCs, ACs and RGCs, we analyzed the retinal transcriptome of *Rbfox2*^*−/−*^ (n = 4) and control (n = 4) animals with RNA-seq. Six month old animals (for *Rbfox2*^*−/−*^ 4 month after tamoxifen-induced *Rbfox2* downregulation) were used in this experiment. Information on Rbfox2-regulated genes, including a complete gene list with fold changes (experimental vs. control), uncorrected and FDR corrected p-values, absolute gene level quantification using FPKM and log count per million (logCPM), as well as top rated differentially regulated genes (DEG) in *Rbfox2*^*−/−*^ animals, are presented in Tables S1 and S2, respectively. Gene set enrichment analysis (GSEA) of the RNA-seq data identified genes involved in RNA metabolic processes, including transcription and splicing, as the top enriched DEG set in *Rbfox2*^*−/−*^ retinal transcriptome compared to control (Table 1). Highly enriched gene sets in *Rbfox2*-deficient retinas also included genes associated with microtubule organization, DNA repair and mitochondrial gene expression (Table 1). The major gene sets/processes that were underrepresented in *Rbfox2*^*−/−*^ retinas include the extracellular matrix, ion transport, neuron differentiation and synapse organization (Table 2). DEGs associated with RNA metabolism are listed in Tables S3 and S4. Expression levels of several DEGs, including bestrophin 2 (*Best2*), glutathione S-transferase alpha3 (*Gsta3*), solute carrier family 13 member 4 (*Slc13a4*), thioredoxin interacting protein (*Txnip*), calmodulin like 3 (*Calml3*), cytochrome P450 monooxygenase (*Cyp4a12b*), olfactory receptor 12 (*Olfr12*) and tumor-associated calcium signal transducer 2 (*Tacstd2*) were arbitrarily chosen for further analysis with real-time PCR to validate RNA-seq data (Fig. 8A). Quantitative PCR data were in agreement with the RNA-seq results. Heatmap of top 100 DEGs (29 downregulated and 71 upregulated genes) identified with EdgeR^32^ (p < 0.01) is shown in Fig. 8B. Major pathways identified with the Kyoto Encyclopedia of Genes and Genomes (KEGG) pathway enrichment analyses^33,34^ of the top 100 DEGs include circadian rhythm, circadian entrainment, dopaminergic synapse, glutamatergic/cholinergic synapse, calcium signaling, PI3K-AKT signaling and protein processing in ER (Table 3).

**Table 1:** Top 50 gene sets and processes overrepresented in *Rbfox2* deficient retinas.

Gene sets and processes	Rbfox2^−/−^ vs. control
RNA_BINDING	5.813049
RNA_METABOLIC_PROCESS	5.6331453
RIBONUCLEOPROTEIN_COMPLEX	5.1632953
RIBONUCLEOPROTEIN_COMPLEX_BIOGENESIS	4.942357
NCRNA_METABOLIC_PROCESS	4.5734477
CHROMOSOME	4.5711336
MRNA_METABOLIC_PROCESS	4.503966
NCRNA_PROCESSING	4.3618264
CATALYTIC_COMPLEX	4.33293
CHROMOSOME_ORGANIZATION	4.317416
DNA_METABOLIC_PROCESS	4.248208
RNA_SPLICING	4.2007804
CATALYTIC_ACTIVITY_ACTING_ON_RNA	4.009043
CILIUM_ORGANIZATION	3.9139876
CILIUM	3.894453
RIBOSOME_BIOGENESIS	3.8750188
RNA_SPLICING_VIA_TRANSESTERIFICATION_REACTIONS	3.8675883
SPLICEOSOMAL_COMPLEX	3.7431524
CHROMOSOMAL_REGION	3.7430563
DNA_REPAIR	3.7317252
TRANSFERASE_COMPLEX	3.7088425
RIBONUCLEOPROTEIN_COMPLEX_SUBUNIT_ORGANIZATION	3.687478
MITOCHONDRIAL_TRANSLATION	3.6841497
MITOCHONDRIAL_GENE_EXPRESSION	3.6695669
RRNA_METABOLIC_PROCESS	3.6613529
CILIARY_PART	3.6463401
MICROTUBULE_CYTOSKELETON	3.593462
TELOMERE_ORGANIZATION	3.5413632
PROTEIN_DNA_COMPLEX_SUBUNIT_ORGANIZATION	3.5132537
NUCLEOLUS	3.4656286
NON_MOTILE_CILIUM	3.4496777
INTRACELLULAR_PROTEIN_TRANSPORT	3.4351099
CELL_CYCLE_PROCESS	3.4279068
NUCLEOPLASM_PART	3.4215302
CELL_CYCLE	3.3388064
CATALYTIC_ACTIVITY_ACTING_ON_DNA	3.3231313
CELLULAR_PROTEIN_CONTAINING_COMPLEX_ASSEMBLY	3.3193235
CHROMOSOME_TELOMERIC_REGION	3.3132815
INTRACELLULAR_TRANSPORT	3.2616508
NUCLEAR_CHROMOSOME	3.2463987
STRUCTURAL_CONSTITUENT_OF_RIBOSOME	3.1385295
RIBOSOMAL_SUBUNIT	3.1363742
DOUBLE_STRAND_BREAK_REPAIR	3.1282332
PROTEIN_FOLDING	3.1267197
9PLUS0_NON_MOTILE_CILIUM	3.1065962
PEPTIDE_BIOSYNTHETIC_PROCESS	3.1017811
CELLULAR_RESPONSE_TO_DNA_DAMAGE_STIMULUS	3.1016924
RNA_CATABOLIC_PROCESS	3.0987551
NON_MOTILE_CILIUM_ASSEMBLY	3.097095

**Table 2:** Top 50 gene sets and processes underrepresented in *Rbfox2* deficient retinas.

Gene sets and processes	Rbfox2^−/−^ vs. control
INTRINSIC_COMPONENT_OF_PLASMA_MEMBRANE	− 6.5516
COLLAGEN_CONTAINING_EXTRACELLULAR_MATRIX	− 6.42253
LOCOMOTION	− 6.16149
EXTRACELLULAR_MATRIX	− 6.15073
CELL_MOTILITY	− 6.13956
BIOLOGICAL_ADHESION	− 5.94402
MOLECULAR_TRANSDUCER_ACTIVITY	− 5.73078
CIRCULATORY_SYSTEM_DEVELOPMENT	− 5.31648
EXTRACELLULAR_MATRIX_STRUCTURAL_CONSTITUENT	− 5.31585
TRANSMEMBRANE_SIGNALING_RECEPTOR_ACTIVITY	− 5.2742
CELL_SURFACE	− 5.12442
ION_TRANSPORT	− 5.0971
POSITIVE_REGULATION_OF_MULTICELLULAR_ORGANISMAL_PROCESS	− 5.03205
ANATOMICAL_STRUCTURE_FORMATION_INVOLVED_IN_MORPHOGENESIS	− 5.02041
REGULATION_OF_CELL_DIFFERENTIATION	− 5.01088
NEGATIVE_REGULATION_OF_MULTICELLULAR_ORGANISMAL_PROCESS	− 5.00931
NEURON_DIFFERENTIATION	− 4.97136
NEUROGENESIS	− 4.94714
REGULATION_OF_CELLULAR_COMPONENT_MOVEMENT	− 4.92124
TUBE_DEVELOPMENT	− 4.84123
BLOOD_VESSEL_MORPHOGENESIS	− 4.83541
CARDIOVASCULAR_SYSTEM_DEVELOPMENT	− 4.78599
RESPONSE_TO_OXYGEN_CONTAINING_COMPOUND	− 4.76589
POSITIVE_REGULATION_OF_DEVELOPMENTAL_PROCESS	− 4.72038
ANIMAL_ORGAN_MORPHOGENESIS	− 4.66922
CELLULAR_COMPONENT_MORPHOGENESIS	− 4.6621
TUBE_MORPHOGENESIS	− 4.59705
RESPONSE_TO_ORGANIC_CYCLIC_COMPOUND	− 4.56083
CATION_TRANSPORT	− 4.55587
REGULATION_OF_NERVOUS_SYSTEM_DEVELOPMENT	− 4.54805
CELL_JUNCTION	− 4.54081
REGULATION_OF_CELL_DEVELOPMENT	− 4.46153
NEURON_DEVELOPMENT	− 4.40799
POSITIVE_REGULATION_OF_CELL_DIFFERENTIATION	− 4.38739
METAL_ION_TRANSPORT	− 4.36452
TAXIS	− 4.33973
RESPONSE_TO_LIPID	− 4.32946
ION_TRANSMEMBRANE_TRANSPORT	− 4.2766
REGULATION_OF_ANATOMICAL_STRUCTURE_MORPHOGENESIS	− 4.27202
REGULATION_OF_TRANSPORT	− 4.27153
ACTIN_FILAMENT_BASED_PROCESS	− 4.26263
RESPONSE_TO_ENDOGENOUS_STIMULUS	− 4.2577
NEGATIVE_REGULATION_OF_DEVELOPMENTAL_PROCESS	− 4.2505
EXTRACELLULAR_STRUCTURE_ORGANIZATION	− 4.21202
ENZYME_LINKED_RECEPTOR_PROTEIN_SIGNALING_PATHWAY	− 4.19864
PLASMA_MEMBRANE_REGION	− 4.1871
PLASMA_MEMBRANE_PROTEIN_COMPLEX	− 4.16876
CELL_MORPHOGENESIS_INVOLVED_IN_DIFFERENTIATION	− 4.15622
CELLULAR_RESPONSE_TO_OXYGEN_CONTAINING_COMPOUND	− 4.1259

**Table 3:** KEGG pathway enrichment analyses of the top 100 DEGs in control vs. *Rbfox2*^*−/−*^ retinal transcriptome.

Pathway	Gene symbol	Gene name	Control/Rbfox2^−/−^
Circadian rhythm	Arntl	Aryl hydrocarbon receptor nuclear translocator-like	+
	Bhlhe41	Basic helix-loop-helix family, member e41	−
	Cry2	Cryptochrome 2 (photolyase-like)	−
	Per1	Period circadian clock 1	−
Circadian entrainment	Grin1	Glutamate receptor, ionotropic, NMDA1 (zeta 1)	−
	Gngt1	G protein, gamma transducing activity polypeptide	+
	Itpr1	Inositol 1,4,5-trisphosphate receptor 1	+
	Per1	Period circadian clock 1	−
Dopaminergic synapse	Atf6b	Activating transcription factor 6 beta	+
	Arntl	Aryl hydrocarbon receptor nuclear translocator-like	+
	Cacna1a	Ca channel, voltage-dependent, P/Q type, alpha 1A	−
	Gngt1	G protein, gamma transducing activity polypeptide 1	+
	Itpr1	Inositol 1,4,5-trisphosphate receptor 1	+
	Scn1a	Sodium channel, voltage-gated, type I, alpha	−
Glutamatergic/cholinergic synapse	Cacna1a	Ca channel, voltage-dependent, P/Q type, alpha 1A	−
	Gngt1	G protein, gamma transducing activity polypeptide 1	+
	Itpr1	Inositol 1,4,5-trisphosphate receptor 1	+
Calcium signaling	Cacna1a	Ca channel, voltage-dependent, P/Q type, alpha 1A	−
	Grin1	Glutamate receptor, ionotropic, NMDA1 (zeta 1)	−
	Itpr1	Inositol 1,4,5-trisphosphate receptor 1	+
	Stim1	Stromal interaction molecule 1	−
PI3K-AKT signaling	Flt1	FMS-like tyrosine kinase 1	−
	Atf6b	Activating transcription factor 6 beta	+
	Gngt1	G protein, gamma transducing activity polypeptide 1	+
	Hsp90b1	Heat shock protein 90, beta (Grp94), member 1	+
	Tnr	Tenascin R	−
Protein processing in ER	Atf6b	Activating transcription factor 6 beta	+
	Calr	Calreticulin (Calr)	+
	Hspa5	Heat shock protein 5	+
	Hspa8	Heat shock protein 8	+
	Hsp90b1	Heat shock protein 90, beta (Grp94), member 1	+
	Hyou1	Hypoxia up-regulated 1	+
	Pdia3	Protein disulfide isomerase associated 3	+
	Ubqln4	Ubiquilin 4	+
Estrogen signaling/thyroid hormone synthesis	Atf6b	Activating transcription factor 6 beta	+
	Hspa5	Heat shock protein 5	+
	Hsp90b1	Heat shock protein 90, beta (Grp94), member 1	+
	Itpr1	Inositol 1,4,5-trisphosphate receptor 1	+
	Hspa8	Heat shock protein 8	+
Antigen processing and presentation	Calr	Calreticulin	+
	Hspa8	Heat shock protein 8	+
	Pdia3	Protein disulfide isomerase associated 3	+

## Discussion

Here we present data on the expression of Rbfox2 in adult and differentiating mouse retinal cells and the effects of *Rbfox2* deficiency on visual function and the retinal transcriptome. This is a continuation of our efforts to characterize the expression and function of Rbfox proteins in the retina; results of a similar work on Rbfox1 were published earlier^16^. We first identified *Rbfox1* and *Rbfox2* in the retina after analyzing gene expression profiles of RGCs^7^. RGCs collect, process and send both image-forming and non-image forming visual information from the retina to target regions in the brain via their axons in the optic nerve. Here we show that in both developing and mature retinas, Rbfox2 expression is restricted to RGCs, HCs and ACs. ACs and HCs are retinal interneurons involved in forming visual signals by feedback and feedforward inhibition. HCs make synaptic connections with photoreceptors and bipolar cells in the OPL and their activity is essential for establishing center-surround receptive-field properties in the visual pathway. ACs form synaptic connections with bipolar cells and RGCs within IPL and by modulating their activity, they shape their receptive fields’ spatial and temporal characteristics^35^. In the mammalian retina, HCs are represented by 2–3 distinguishable subtypes, whereas there are more than 30 morphological and functional subtypes of ACs and RGCs. In mouse retinal sections that were immunostained with Rbfox2 antibodies and markers for HCs, ACs and RGCs we observed colocalization of Rbfox2 with all HCs and RGCs. With respect to Rbfox2 expression in ACs, the vast majority of dACs as well as 2–3 rows of cells in the INL adjacent to IPL were Rbfox2^+^. Several markers for AC subtypes, including GABA, ChAT, NPY, EBF1, GlyT1 and vGlut3 were used to identify Rbfox2^+^ ACs. All GABAergic ACs in the GCL and the vast majority of GABAergic ACs in the INL express Rbfox2. Almost all Rbfox2^+^ ACs are GABAergic. A subtype of GABAergic ACs, SACs, were all positive for Rbfox2 in both the INL (type a) and GCL (type b). Extensive overlap of Rbfox2 with NPY and EBF1 expression was also observed, although there were very few Rbfox2^+^ cells that had no staining for NPY or EBF1. Among vGlut3^+^ cells, some that were sparsely distributed in the INL were also stained with Rbfox2. Interestingly, a large number of vGlut3^+^/Rbfox2^+^ cells were observed in the GCL. vGlut proteins, which promote the uptake of glutamate into synaptic vesicles to support synaptic transmission, are represented in the retina by three members: vGLUT1 is localized to photoreceptor and bipolar cell terminals, vGLUT2 is restricted to HCs and RGCs and vGlut3 is expressed in a sparse population of glycinergic ACs. In mouse and rat retinas vGlut3 immunolabeled cells were present in the INL^36,37^. However, in human retinas vGlut3 was detected in the GCL and was colocalized with neurofilament 200, a marker for RGCs^34^. Here we show that vGlut3 immunostaining identifies both sparsely populated ACs in the INL as well as a large number of Rbpms-labeled RGCs in the GCL.

The expression pattern of Rbfox2 in the retina shares similarities with that of Rbfox1: both proteins are present in all RGCs and almost all Rbfox1^+^ and Rbfox2^+^ ACs are GABAergic ACs. However, there are notable differences: (a) Rbfox2 but not Rbfox1 is expressed in HCs; (b) Rbfox1 expression in the INL appears to be restricted to ACs that are located in the innermost layer of the INL, whereas Rbfox2-expressing cells are present in several rows occupied by ACs; and (c) Rbfox2 is expressed in a wider population of dACs in the GCL than Rbfox1. Quantitative analysis of Rbfox2^+^ cells in whole-mounted retinas supports the data observed in cross section: virtually 100% of RGCs are positive for Rbfox1 and, although most dACs are positive for both Rbfox2 and Rbfox1, approximately 6–7% of dACs express Rbfox2 only.

We evaluated Rbfox2 expression during retinal development at E12, E15, P0, P5, P10, P12, P14, P15 and P21. These stages were chosen based on the timeline of retinal cell differentiation and establishment of synaptic connections between them^38^. Expression of Rbfox2 can be seen in differentiating retinal cells at E12. It has been reported that GCL cells born before E11.8 and E12.8 were 98% and 99% RGCs, respectively, and those born after E15.8 were 97% dACs^39^. This suggests that the Rbfox2^+^ cells that we observed at E12 are almost exclusively RGCs. By P0, although the retina was not fully developed yet, the expression pattern of Rbfox2 resembles that of the mature retina. Subcellularly, Rbfox2 appears to be localized predominately to the nucleus irrespective of phases of retinal development. This is different from Rbfox1 subcellular localization, which shifts from cytoplasmic at E12 to predominantly nuclear at P0 and thereafter^16^. The transition in Rbfox1 subcellular distribution coincides with the stage II spontaneous retinal waves of excitation^40^ and can be explained by increased expression of the nuclear Rbfox1 isoform necessary for regulation of the alternative splicing of target genes at this stage of retinal development.

The role of Rbfox2 on retinal morphology and visual function was evaluated in Rbfox2^−/−^ animals. Downregulation of *Rbfox2* in adult animals had no effect on gross retinal architecture or retinal cell morphology. The PLR and depth perception were analyzed in *Rbfox2*^*−/−*^ animals for potential abnormalities in visual function. The PLR helps adapt vision to different levels of light intensity by regulating the pupil diameter. Retinal cells that mediate PLR are intrinsically photosensitive retinal ganglion cells (ipRGCs), the melanopsin-expressing cells that comprise ~ 0.2% of the total number of RGCs^41^. The PLR test of *Rbfox2*-deficent mice showed no detectable abnormalities, suggesting that *Rbfox2* downregulation has no significant effect on the function of ipRGCs. The functional integrity of the retino-geniculo-cortical pathway in *Rbfox2*^*−/−*^ animals was evaluated by analyzing their depth perception. Unlike control animals, *Rbfox2*^*−/−*^ mice exhibit no clear preference for the shallow side and as a group spent almost 3 times more time on the deep side compared to controls. It is noteworthy that depth perception impairment was also observed in *Rbfox1*^*−/−*^ animals, suggesting that the molecular mechanisms underlying this phenomenon caused by downregulation of the *Rbfox1* and *Rbfox2* genes are similar.

A significant overlap in Rbfox1 and Rbfox2 expression in the retina and the fact that these RNA binding proteins recognize the same *(U)GCAUG* element within their target genes, suggest that they may be involved in regulation of same set of genes. This appeared redundancy may explain why a deletion of a single gene, *Rbfox1* or *Rbfox2*, has no dramatic effect on overall retinal morphology. The notion that Rbfox1 and Rbfox2 regulate a similar set of genes is also supported by our observation that animals deficient in either *Rbfox1* or *Rbfox2* have depth perception impairment. Interestingly, the deletion of the third member of Rbox family, *Rbfox3*, which is normally expressed in most types of RGCs, some types of ACs and HCs, resulted in the reduction of IPL thickness, but the number of RGCs and ACs was normal. *Rbfox3*^*−/−*^ mice had normal PLR and optomotor response, leading authors to conclude that RBFOX3 is dispensable for visual function^42^. Although, these observations imply that Rbfox proteins are redundant and may substitute each other, the differences that we observed in their expression pattern suggest that each of these proteins may have distinct functions during retinal development, as well as in mature retinas.

To identify Rbfox2-regulated genes, we analyzed *Rbfox2*^*−/−*^ and control retinal transcriptome. KEGG pathway enrichment analysis identified Rbfox2-regulated genes involved in circadian rhythm, circadian entrainment, dopaminergic synapse, glutamatergic/cholinergic synapse, calcium signaling, PI3K-AKT signaling and protein processing in ER. Regulation of genes involved in synaptic function was expected since Rbfox proteins have been implicated in neuronal differentiation and synaptic transmission in particular^6,27,43^. However, to our knowledge, the coordinated regulation of several core circadian rhythm proteins, including aryl hydrocarbon receptor nuclear translocator-like (Arntl/Bmal1), basic helix-loop-helix family, member e41 (Bhlhe41/Dec2), cryptochrome 2 (Cry2), period circadian clock proteins 1 and 2 (Per1 and Per2), by Rbfox2 is observed for the first time. Expression of these core clock components together with CLOCK play a critical role in cellular rhythm generation. The mechanisms for circadian clock regulation involve transcription-translation feedback loops that self-regenerate with a ~ 24-h rhythm. In mammalian cells, the CLOCK and BMAL1 transcription factors control the expression of *Per1/2* and *Cry1/2*, which in turn inhibit CLOCK and BMAL1^44,45^. These four factors regulate the expression of thousands of clock-controlled genes that coordinate the oscillation of cellular metabolism and physiology. Clock genes are linked directly to metabolic syndromes; polymorphisms circadian clock genes *CLOCK*, *BMAL1*, *Per1/2* and *Cry1/2* have been associated with metabolic disorders, including neuropsychiatric or neurodegenerative diseases^46^. In *Rbfox2*^*−/−*^ retinas, *Arntl/Bmal1* is downregulated, whereas *Cry2* and *Per1/2* are upregulated. The expression of Clock, Bmal1, Cry2 and Per1 in mouse retinas have been shown to be predominantly localized to RGCs, ACs and HCs^47^. These cells, as we described in this study, also express Rbfox2, further collaborating the results of RNA-seq data analysis on possible regulation of core circadian clock genes by Rbfox2.

In summary, Rbfox2 expression in mouse retinas is restricted to cells in the GCL and INL. Virtually all types of RGCs and dACs in the GCL were positive for Rbfox2. In the INL, HCs and 2–3 rows of cells proximal to IPL were also Rbfox2^+^. Among AC subtypes, Rbfox2 is expressed in GABAergic cells, including NPY^+^, EBF1^+^, Vglut3^+^ and ChAT^+^ ACs. There is a significant overlap between Rbfox1 and Rbfox2 expression among RGCs and ACs. However, Rbfox2 is more widely distributed among AC subtypes compared to Rbfox1. In the developing retina, Rbfox2 is present as early as E12 and, unlike Rbfox1, it was localized in the nucleus throughout the retinal differentiation. Downregulation of *Rbfox2* in adult animals had no effect on retinal architecture, however depth perception in these animals had significant deficiency. Transcriptome analysis identified Rbfox2-regulated genes associated with circadian rhythm/circadian entrainment pathways and dopaminergic/glutamatergic/cholinergic synapse function.

## Methods

### Animals

All experiments with animals were approved by the Animal Research Committee of the University of California at Los Angeles and were performed in compliance with the National Institutes of Health Guide for the Care and Use of Animals and the Association for Research in Vision and Ophthalmology Statement for the Use of Animals in Ophthalmic and Vision Research. Wild type (C57BL/6J) and transgenic mice were housed in a room with an ambient temperature of 25 °C, 30–70% humidity, a standard 12/12-h light/dark cycle, with food and water provided ad libitum. Carbon dioxide (CO_2_)-induced asphyxiation was used to euthanize animals. *Rbfox2*^*fl/fl*^ containing LoxP sites flanking *Rbfox2* exons 6 and 7 were kindly provided by Dr. Douglas Black, UCLA^48^.

To generate *Rbfox2*^*−/−*^ animals, *Rbfox2*^*fl/fl*^ homozygous transgenic mice were crossed with *Tg(UBC-cre/ERT2)1Ejb* mice (Jackson Laboratory, Bar Harbor, ME). The resulting heterozygous *Rbfox2*^*fl/*+^*; UBC-Cre*^+*/−*^ mice were crossed with *Rbfox2*^*fl/fl*^ mice to obtain homozygous *Rbfox2*^*fl/fl*^*/UBC-Cre*^+*/−*^ animals. Genotyping for the *Rbfox2*^+*/−*^ and *Rbfox2*^*fl/fl*^ alleles was performed by standard PCR with primers Rbfox2-F (5′-AACAAGAAAGGCCTCACTTCAG) and Rbfox2-R (5′-GGTGTTCTCTGACTTATA CATGCAC). The expected PCR product sizes were: a 323 base pair (bp) for *Rbfox2*^+*/*+^, ~ 400 bp and 323 bp for *Rbfox2*^+*/−*^ and a ~ 400 bp for *Rbfoxl*^*fl/fl*^*.* The presence of *Ubiquitin-Cre* was evaluated with primers Cre-F (5′-GACGATGCAACGAGTGATGA) and Cre-R (5′-AGCATTGCTGTCACTTGGTC) that would produce a PCR fragment with an expected size of 300 bp. Cre recombinase-mediated recombination was induced in adult (~ 2 months old) homozygous *Rbfox2*^*fl/fl*^; *UBC-Cre*^+*/−*^ animals by administration of 200 mg/kg tamoxifen (Sigma, St. Louis, MO) dissolved in corn oil (Sigma) for five consecutive days. Tamoxifen or corn oil (vehicle) was administered by oral gavage. To prevent tamoxifen cross-contamination, tamoxifen-treated mice were housed individually^49^. Experimental animals were viable, had a normal growth rate and behaviorally had no apparent anomalies compared to control animals. Age-matched heterozygous *Rbfox2*^*fl/*+^ mice were used as controls.

### Immunohistochemistry

Immunohistochemistry was performed according to a standard procedure^50^. Briefly, enucleated eyes were fixed with ice-cold 4% paraformaldehyde and cryoprotected in 30% sucrose. Retinal Sects. (14-µm thick) were cut with cryostat, incubated with blocking solution (20% fetal calf serum, 5% goat serum, 0.1% Triton X-100 in PBS) for 30 min. Sections were then incubated with primary antibodies overnight at 4 °C, washed three times with 0.1% Triton X-100 in PBS, incubated with secondary antibodies for 1 h at room temperature, washed again three times, mounted with mounting medium containing DAPI reagent and imaged using a confocal laser scanning microscope Olympus FV3000 (Olympus, MA). The following primary antibodies were used: rabbit Rbfox2, 1:1500 (Bethyl Laboratories, Montgomery, TX); mouse Rbfox1, 1:200 (Novus Biologicals, Littleton, CO); rabbit Rbpms, 1:500^46^; rabbit calbindin D-28K, 1:500 (EMD Millipore, Billerica, MA); rabbit calbindin D-28K, 1:500 (C2724, Sigma); rabbit GABA, 1:2000 (Sigma); goat ChAT, 1:200 (EMD Millipore); rabbit NPY, 1:1000 (Abcam), guinea pig vGlut3, 1:2500 (EMD Millipore), rabbit EBF, 1:2000 (EMD Millipore). The following secondary antibodies were used: Alexa Fluor 568-conjugated donkey anti-mouse IgG, Alexa Fluor 488-conjugated donkey anti-mouse IgG, Alexa Fluor 568-conjugated donkey anti-rabbit IgG, Alexa Fluor 488-conjugated donkey anti-rabbit IgG, Alexa Fluor 568-conjugated donkey anti-guinea pig IgG, Alexa Fluor 647-conjugated donkey anti-guinea pig IgG, Alexa Fluor 488-conjugated donkey anti-goat IgG (all 1:500; Life Technologies, Carlsbad, CA), Alexa Fluor 488-conjugated goat anti-rabbit IgG, 1:500 Thermo Fisher Scientific, Canoga Park, CA; Alexa Fluor 568-conjugated goat anti-mouse IgG, 1:100 (Thermo Fisher Scientific). Images were acquired with an Olympus FV3000 confocal laser scanning microscope (Olympus, MA). Retinas from at least three animals were used for each immunostaining experiment.

### Quantification of Rbfox2^+^ cells in whole-mounted retinas

Cell quantification was performed as described earlier^16,50^. The retinas were dissected from enucleated eyeballs, fixed in 4% paraformaldehyde in 0.1 M phosphate buffer, incubated with 10% serum for 1 h to block nonspecific staining, and then with primary antibodies overnight at 4 °C. After incubation with secondary antibodies overnight at 4 °C, the retinas were mounted flat with several radial cuts on a glass slides with the GCL facing upward. The retinas were divided into four quadrants: superior, inferior, nasal and temporal and four sampling fields (0.31 × 0.31 mm each) were imaged at 0.5 mm from the center of the optic nerve in each retina. Quantification of Rbfox2^+^ cells was performed by counting Rbfox2^+^/Rbpms^+^ and Rbfox2^+^/Rbfox1^+^ cells. Three retinas were used for each immunostaining experiment. Data are presented as the mean ± SEM.

### PLR video recordings and analysis

PLR was performed during the light phase of the light–dark cycle. Animals were dark-adapted for 1 h before the experiment. For PLR recording mice were restrained with a head post similar to that described by Cahill and Nathans^51^. Animals were exposed to a 30-s stimulus of a NIR-100 light source. Video recordings were obtained by six infrared digital cameras (three per eye). Screen shots of the video were generated every 5 s with VLC Media Player for 1 min video duration. The pupil area was measured with ImageJ software (NIH) and normalized to baseline. PLR for each animal was recorded and analyzed in three independent experiments.

### Visual cliff test

A visual cliff test was used to evaluate depth perception in *Rbfox2*^*−/−*^ animals. The test was performed as described earlier^16,31^. Briefly, a box with a transparent bottom was placed on the edge of the table (“shallow” side) so half of it was suspended above the floor (“deep side”). The illusion of the cliff was created by covering the table and the floor with 1-in. squared black checker linoleum. Both sides of the setup were equally illuminated. The time the animal spent on the shallow and deep sides during 5 min of testing was recorded. Each mouse was tested five times. To eliminate tactile placing responses that may interfere with testing of visual function, the vibrissae of the animals were removed. The glass and central platform were thoroughly cleaned after each test. Six control and ten *Rbfox2*^*−/−*^ animals were used in this experiment. Data are presented as the mean ± SD.

### RNA sequencing (RNA-seq) analysis of retinal transcriptome

Retinas from *Rbfox2*^*−/−*^ (n = 4) and control (n = 4) animals were dissected at approximately 10 am. Total retinal RNA was isolated with an RNeasy mini kit (Qiagen, Germantown, MD). Libraries for RNA-seq were prepared with Nugen Universal Plus mRNA-seq library preparation kit (Tecan Genomics, Redwood City, CA). The workflow consisted of mRNA enrichment with poly(A) selection, RNA fragmentation, cDNA generation and end repair, and followed by adaptor ligation and PCR amplification. Sequencing was performed on a NovaSeq 6000 (Illumina, San Diego, CA) for 2 × 150 run. Data quality checks were done on an Illumina SAV. Demultiplexing was performed with the Illumina Bcl2fastq2 v 2.17 program. Reads were aligned to the latest mouse mm10 reference genome with the Spliced Transcripts Alignment to a Reference (STAR) software. Total counts of read-fragments aligned to known gene regions within the mouse mm10 refSeq reference annotation were used as the basis for the quantification of gene expression. Fragment counts were derived using the HTS-seq program with the mm10 Ensembl transcripts as a model. Various QC analyses were conducted to assess the quality of the data and to identify potential outliers. The number of total reads ranged from 218.5 to 290.2 M (average 249.5 M). Uniquely mapped reads were 91.75% to 92.37% (average 92.10%). Only uniquely mapped reads were used for subsequent analyses. Differentially expressed genes (DEG) were identified with three bioconductor packages, edgeR, limma + voom and limma, which were then considered and ranked based on the False Discovery Rate (FDR Benjamini Hochberg adjusted p-values) and simple p-values. The Kyoto Encyclopedia of Genes and Genomes (KEGG) pathway enrichment analyses of the DEGs were performed with the Database for Annotation, Visualization, and Integrated Discovery (DAVID) (https://david.ncifcrf.gov/). RNAseq data has been deposited within the Gene Expression Omnibus (GEO) repository (http://www.ncbi.nlm.nih.gov/geo), accession number GSE155411.

### Quantitative real-time PCR

Real time PCR was performed according to a standard protocol^16^. Mouse retinal total RNA was extracted with RNeasy mini kit (Qiagen) according to the manufacturer’s protocol. First-strand cDNA synthesized with SuperScript III First-Strand Synthesis System (Thermo Fisher Scientific), primers (Table 4) and the SYBR Green PCR Master Mix (Applied Biosystems/Life Technologies) were used for real-time PCR. The LightCycler 480 II (Roche Applied Science, Mannheim, Germany) was used for cycling and real-time quantitative detection of PCR products. Expression levels of target genes were normalized to the threshold cycle (Ct) of *GAPDH*. The expression level of each gene was calculated relative to the expression of the control group: 2−∆∆Ct, where ∆∆Ct = Exp (Ct, target – Ct, *GAPDH*) – Ctrl (Ct, target – Ct, *GAPDH*).

**Table 4:** Primers used for quantitative real-time PCR.

Gene	Forward	Reverse
*Best2*	5′-ACC CCT ACG GAC ACC TAC CTA AT	5′-TGA GGG AAG CAG CAA CAG CTG
*Calml3*	5′-CAT GAG GCT TCT GGG CTA CAA	5′-GCC AGA ATA AGA TGC AGA AAG TGA
*Cyp4a12b*	5′-TCA CAG AAC AAG ACA GCT CAA ATG	5′-GGG TGG AGG TGA ACA AGG TGA
*Gsta3*	5′-TCA GGC AAC TAT AAG TAC ATA GC	5′-GAA TTG ACA CAG ACG CCT TAG A
*Olfr12*	5′-GGA ATG CTT CTG TGT AGA TCA CTG	5′-GGT AAC TGA GTC CCA CAC TTT CG
*Slc13a4*	5′-AGC GGA CAG TTA CCA AGC ACA G	5′-GTA GGC TTT CTT GAG AGT TGG TG
*Txnip*	5′-TGA AGC ATC TGT ATT AGC GCA TT	5′-GCT GGG GTG TAC CCG TTC
*Tacstd2*	5′-CTC GGC ACC TCA GAC CAG ATG	5′-AGC TCA GCA TCT AGA GAA CTT GTT

### Statistical analysis

Statistical analysis was performed as described earlier^16^. For cell quantification, an unpaired Student’s t-test was used. P < 0.05 was considered statistically significant. For the behavior test, a repeated measures ANOVA model was used to analyze the mean difference between the two groups in time spent on the deep side, after controlling for repeated measurements within each animal.

## Supplementary information

Supplementary Table S1.

Supplementary Table S2.

Supplementary Table S3.

Supplementary Table S4.

## Data Availability

All data associated with this study are included in the paper or its supplementary information file.
